# Supplementary material for: Field deployment of a mobile suitcase laboratory for Buruli ulcer diagnosis in Ghana
Source: PLoS Negl Trop Dis. 2026 May 4;20(5):e0013683. doi: 10.1371/journal.pntd.0013683 (PMC13160437; doi:10.1371/journal.pntd.0013683)
Supplement: S1 Table — (DOCX) [file pntd.0013683.s003.docx]

**S1 Table. Comparison of molecular diagnostic platforms for Buruli ulcer detection**

| **Feature** | **qPCR (Reference Laboratory)** | **Portable qPCR ( Biomeme)** | **LAMP** | **RPA** |
| --- | --- | --- | --- | --- |
| Target | IS2404 | IS2404 | IS2404 | IS2404 |
| Amplification method | Thermal cycling PCR | Thermal cycling PCR | Isothermal (LAMP) | Isothermal (RPA) |
| Time to result | ≥24–48 h | Yet to be determine | ~60–75 min | ~45 min |
| Amplification time | ~1.5–2 h | ~45–60 min | ~30–60 min | ~15 min |
| DNA extraction | Standard laboratory kits | Proprietary kits | Variable (often simplified methods) | Rapid GenoLyse-based method |
| Equipment requirements | Full laboratory infrastructure | Portable suitcase/laboratory with minimal space/equipment | Portable suitcase/laboratory with minimal space /equipment | Portable suitcase/ laboratory with minimal space /equipment |
| Power requirements | Stable electricity required | Battery-powered options available | Low (simple heating device) | Battery/solar-powered (portable power pack) |
| Infrastructure needs | High (centralized laboratory) | Low to moderate | Low (field-deployable) | Low (field-deployable) |
| Technical expertise | Highly trained personnel | Moderately trained personnel | Moderately trained personnel | Moderately trained personnel |
| Sensitivity | ~95–100% | ~97–100% (laboratory conditions) | ~70–90% (field reports vary) | ~69-95% (field reports) |
| Specificity | ~100% | ~94–100% | ~35–75% (reported variability) | 100% |
| Cost per test* | ~$25 | ~$15–20 | ~$5–15 | ~$10 |
| Initial setup cost* | Unknown | Moderate–high | Unknown | $10,000 (including power system) |
| Portability | No | Yes | Yes | Yes |
| Suitability for low-resource settings | Limited | High | High | High |
| Field readiness | No | Yet to be used in field settings | Moderate (used in field settings) | Promising (requires further optimization) |

*****Cost is substantially influeunce by both global and local economic conditions.
