# Supplementary material for: Field deployment of a mobile suitcase laboratory for Buruli ulcer diagnosis in Ghana
Source: PLoS Negl Trop Dis. 2026 May 4;20(5):e0013683. doi: 10.1371/journal.pntd.0013683 (PMC13160437; doi:10.1371/journal.pntd.0013683)
Supplement: Attachment — Submitted filename: Response to review.pdf [file pntd.0013683.s006.pdf]

Kumasi Centre for Collaborative Research in Tropical Medicine KCCR

KCCR
KNUST, UPO
Kumasi, Ghana
Tel.: + 233 3220 60351
Fax: +233 3220 62017
info@kccr.de

9th April, 2026

Dear Editor,

We thank all reviewers for their constructive and insightful comments. We have carefully revised the manuscript accordingly. Below, we provide a detailed point-by-point Response.

Authors Responses to reviewers

Reviewer #3:

1. Fig 4: The background of this figure is too dark, the axis description for the y-axis is missing (assuming it is minutes?)

Response:

Thank you for this observation. We have revised Figure 4 by improving the background contrast and adding the missing y-axis label (“turn around time (minutes”)) to enhance readability.

2. Thank you to the authors for considering the feedback and adapting the manuscript accordingly. There are still some minor changes needed (e.g. adapting the readability of Figure 4), but the quality of the manuscript has increased substantially.

Response:

We appreciate the reviewer’s positive feedback and are pleased that the revisions have improved the manuscript.

3. line 143: seventy-three instead of seventy- three

Response:

This has been corrected in line 152.

4. line 183: sentences do not fit together

Response

We have revised this section for clarity and improved sentence flow.

5. line 273: remove b at end of line

Response

This has been Corrected.

6. IS2404 is a multi-copy target, could you indicate to how many bacteria the detection limit of 45 copies corresponds to?

Response:

IS2404 is indeed a multi-copy insertion sequence (~200 copies per genome of *Mycobacterium ulcerans*). Therefore, a detection limit of 45 copies corresponds approximately to **<1 bacterial genome equivalent**. We have clarified this in the manuscript.

7. it is still not clear if the samples for field diagnosis and qPCR were taken from the same lesion, please indicate in the methods

Response:

We have clarified in the Methods section that **two separate samples were collected from the same lesion**; one for RPA (field testing) and one for qPCR.

8. line 343: How do the authors expect the costs to reduce by a 10-fold?

Response:

We have expanded this section to clarify that the projected cost reduction could be achieved through: bulk production of reagents, local manufacturing, simplified extraction methods and increased market competition.

9. The authors could indicate more clearly in the conclusions that this promising setup still needs some tweaking before large-scale implementation

Response:

We agree and have revised the conclusion to explicitly state that **further optimization and large-scale validation are required before widespread implementation**.

Reviewer #4:

10. The discussion could briefly clarify the operational advantages of the RPA-based platform relative to previously described portable molecular diagnostic systems, particularly portable qPCR platforms referenced in the manuscript.

Response

We have expanded the Discussion to clarify advantages of RPA, including: lower energy requirement, faster amplification (~15 minutes), no need for thermal cycling and ability to incoperate other detection method such as laterial detection and simpler instrumentation.

11. The authors may consider briefly expanding the discussion on potential strategies to improve sensitivity for FNA samples.

Response:

We have added discussion points including improving DNA extraction efficiency, sample dilution strategies to reduce inhibitors and optimizing sample collection techniques.

12. A short statement highlighting the level of training required for personnel to operate the mobile laboratory platform would further strengthen the implementation perspective.

Response:

We have added a statement indicating that current operation requires trained molecular personnel, but with structured training, the platform could be operated by healthcare workers or field staff.

Reviewer #5:

13. The abstract reports overall sensitivity and specificity but does not indicate that diagnostic performance differed between sample types (swab vs. FNA). Consider briefly mentioning this difference to provide a more balanced summary.

Response:

We have revised the abstract to briefly indicate that **sensitivity differed between swab and FNA samples.**

14. Please clarify the specific advantages of RPA over other field-friendly molecular methods such as LAMP or portable PCR platforms.

Response:

The specific advantages of RPA has been discussed in the discussion section. A Brief statement of this has been mention in the introduction also. RPA is faster reaction time

than LAMP and has lower equipment complexity than PCR.

15. The study design should be explicitly stated in the Methods section.

Response:

We have explicitly stated the study design as a **prospective field-based diagnostic evaluation study** in the Methods section.

16. Please describe the DNA extraction method and the PCR procedure used as the gold standard.

Response:

Detailed descriptions of the **extraction method**, and the **IS2404 qPCR protocol used as gold standard** have been added.

17. Please clarify whether PCR and RPA were performed on the same specimen or on separate samples collected from the same lesion.

Response:

We have clarified in the Methods section that **two separate samples were collected from the same lesion**; one for RPA (field testing) and one for qPCR.

18. Since PCR and RPA results are available for the same participants, the authors may consider reporting an agreement analysis (e.g., McNemar test or kappa statistic).

Response:

We appreciate this suggestion. We have now included an **agreement analysis (Cohen's kappa)** to assess concordance between RPA and PCR.

19. Please clarify how the turnaround time was determined.

Response

We clarified that turnaround time was measured from: **sample collection to completion of amplification/result readout.**

20. Please clarify how the assay cost was calculated and what components were included.

Response:

The assay cost was calculated based on reagents, consumables and extraction materials (excluding capital equipment costs).

21. Patient demographic characteristics should be clearly presented.

Response:

Demographic data are presented and referenced in the Results section (Table 2).

22. The sample size calculation required 49 PCR-positive cases, but only 39 PCR-positive cases were included. This deviation should be acknowledged as a limitation.

Response

We have acknowledged that only **39 PCR-positive cases** were included instead of 49 and this is now explicitly discussed as a limitation.

23. If positivity rates differed between PCR and RPA, this should be explicitly reported.

Response:

We clarified and explicitly reported differences in positivity rates between PCR and RPA.

24. The subgroup sensitivity analyses (particularly for FNA samples) are based on relatively small sample sizes, resulting in wide confidence intervals. The authors may consider reporting positivity rates instead.

Response:

We agree and have emphasized wide confidence intervals additionally reported **positivity rates** for clarity.

25. The reasons why the RPA assay demonstrated lower sensitivity compared with the previous study and the Biomeme system should be discussed.

Response:

We expanded the discussion to include possible reasons field conditions, crude DNA extraction, sample variability and use of separate specimens from the same lesion.

26. The reported $10 per test cost requires clarification regarding the cost components included in this estimate.

Response:

We clarified that the estimate includes RPA reagents, extraction kits, consumables and excludes equipment and personnel costs.

27. Tables / Presentation:.A head-to-head comparison table of PCR, Biomeme, and the RPA assay (e.g., test cost, time to result, setup cost, infrastructure requirements, and operating conditions) would help clarify the comparative advantages of the proposed platform.

Response:

We have added a **new comparison table** summarizing cost per test, turnaround time, infrastructure requirements and portability.

28. The manuscript contains several typographical and grammatical errors (e.g., “laboratoy,” “polymerse,” “diganosising”). Careful proofreading is recommended.

Response:

The manuscript has undergone **thorough proofreading**, and all identified errors have been corrected.
