# Supplementary material for: Field deployment of a mobile suitcase laboratory for Buruli ulcer diagnosis in Ghana
Source: PLoS Negl Trop Dis. 2026 May 4;20(5):e0013683. doi: 10.1371/journal.pntd.0013683 (PMC13160437; doi:10.1371/journal.pntd.0013683)
Supplement: Attachment — Submitted filename: Authors response to reviewers.docx [file pntd.0013683.s005.docx]

Dear Editor,

We would like to thank the reviewers for the helpful comments. Based on these suggestions, we have revised the manuscript.

Please find our point-by-point response below. Changes are highlighted in yellow in the revised manuscript and tracked.

.

**Authors responses to journal requirements**

Potential Copyright Issues:

I. We took the pictures ourselves, and the individuals have agreed (written consent form) to their photos being published.
II. The map in the manuscript was drawn by us, and we have provided the location and the source of the basemap shapefile onto which data was plotted.

Reviewer(s)' Comments to Author:

Reviewer #1:

1. There is a field friendly tool available which has been published from which the first author is the last author of the current proposed manuscript (<https://pubmed.ncbi.nlm.nih.gov/37228126/>) .
   This article weakens the claim of originality, as it already presents a field-friendly molecular confirmation method for BU. Although the previously published tool relies on qPCR and the current study focuses on RPA, two different amplification technologies with distinct operational formats,they ultimately serve the same purpose. Therefore, the added value of the new RPA-based test must be clearly articulated in the manuscript, and a direct comparison with the published qPCR method should be included. This is currently missing and should be addressed to demonstrate the specific advantages and justification for the new approach. The current data and analyses do not yet fully support the claims, as the technical validation of the assay is missing and only the in-field evaluation is presented. I recommend consulting the MIQE guidelines to identify the essential technical experiments that should be added, including determination of the limit of detection (LOD), technical reproducibility, repeatability, and additional control reactions. The inclusion of the EQA BU LabNet panel would further strengthen validation and provide an external benchmark. Several necessary controls are also lacking: sampling controls, DNA extraction controls (both positive and negative), and RPA assay controls (positive and negative). These should be incorporated to ensure assay reliability and to meet standard reporting expectations. Given the existence of a the Biomeme Franklin Mobile qPCR for rapid detection of M. ulcerans, a direct comparison is needed. This includes comparing both the detection method and the rapid DNA extraction solution (Genolyse vs Biomeme M1 cartridges) against the rapid extraction protocol proposed here. A side-by-side comparison of DNA extraction methods using the standard IS2404 qPCR as the reference outcome would provide robust evidence for the added value of the new method. A cost comparison covering both the DNA extraction workflow and the RPA/Biomeme detection approaches should be included to clearly position the practicality and field relevance of the proposed solution.
   Also the number of steps can be included as a comparison , as each additional step is a possible chance of contamination , which is something you would like to avoid when you are molecularly confirming cases. Additinoaaly it would be helpful to also mentioned the swabs that were used. these can give a tremendous difference, cotton swab are preferably not used but floqswab are perfect. they release the capture pathogens way better then cotton swabs.

**Response:
We appreciate the reviewer's important comment. We recognize the previously published field-friendly qPCR assay (Biomeme Franklin Mobile qPCR), which we discuss in the manuscript regarding its sensitivity, specificity, and cost. However, this platform has not yet been implemented in routine field settings for Buruli ulcer diagnosis. The primary objective of our study is to evaluate the field implementation and operational feasibility of an analytically validated RPA assay. While both assays serve the same diagnostic purpose, they utilize fundamentally different amplification technologies and operational formats. Both are aligned with the WHO's priority of developing multiple field-adapted molecular tools to enable rapid Buruli ulcer diagnosis in resource-limited settings. We compared the RPA assay against the gold-standard in-house IS2404 qPCR, which remains the accepted reference method, rather than the Biomeme platform, due to different study timelines. Importantly, we have comprehensively established the analytical sensitivity, specificity, limit of detection, reproducibility, and repeatability of the RPA assay in our previous work [1]. The current study focuses on real-world field deployment (in mobile suitcase), including the incorporation of appropriate extraction and assay controls. We agree that direct side-by-side comparisons of RPA and portable qPCR platforms, along with rapid DNA extraction methods and cost analyses, would strengthen the evidence base and are planned for future studies. Lastly , cotton swabs are currently in used in our setting, this has been mentioned in the method section.**

2. Are the results clearly and completely presented?yes, but raw data from RPA should be added in the article or in supplemental

**Response:**

**A supplementary file containing all raw data has been added to the manuscript.**

3. Nice overview of the suitcase; however, the picture quality should be optimized.

**Response:**

**We have tried to optimize the picture quality.**

4. Step-by-step protocol are not included limitation are not mentioned

**Response:**

**We have revised the methods section to include a clearer, description of the BU-RPA mobile laboratory workflow to improve transparency and reproducibility. We have provided the link to our protocols (protocols.io) In addition, the limitations of the study are discussed in the discussion section.**

Reviewer #2:

The manuscript presents an innovative approach to improving Buruli ulcer (BU) diagnosis through a mobile suitcase laboratory using Recombinase Polymerase Amplification (RPA). The study addresses a critical gap in point-of-care molecular diagnostics for neglected tropical diseases (NTDs) and demonstrates feasibility in field conditions. The work is relevant, timely, and potentially impactful for resource-limited settings. However, some areas require clarification, refinement, and additional detail to strengthen the manuscript.

5. Title: Deployment of a mobile suitcase laboratory for field diagnosis of Buruli ulcer disease in Ghana
   Shorten for clarity and impact:
   Suggested: “Field deployment of a mobile suitcase laboratory for Buruli ulcer diagnosis in Ghana.”

**Response:**

**We thank the reviewer for the suggestion and have according changes the title of the manuscript as “Field deployment of a mobile suitcase laboratory for Buruli ulcer diagnosis in Ghana.”**

6. Clarify whether the two samples used for both Mu-RPA and PCR were collected at the same site of the lesion or from different sites. This may affect interpretation of sensitivity and specificity.

**Response:
Two samples were taking from the the same site and one sample was used for each assay. This has been clarified in the method section.**

7. Add details on contamination control measures and internal quality controls.

**Response:**

**Three controls were included in the test. An extraction control and a "no template control" (NTC) were added to the reaction to detect any contamination of reagents or equipment by extraneous nucleic acids, ensuring that results are not influenced by false positives. Additionally, a positive control consisting of extracted DNA from M. ulcerans was included to verify that the RPA system, comprising reagents, primers, polymerase, and amplification machine, was functioning correctly.**

8. Specify operator training level and discuss feasibility for non-specialists.

**Response:**

**In this study, the BU-RPA mobile laboratory platform was operated by a single, highly trained individual with advanced expertise in molecular diagnostics. This has been clearly specified and discussed as a limitation in the discussion section and highlighted the need for structured training and simplified workflows to enable future implementation by non-specialist healthcare workers in low resource settings.**

9. How did authors manage contentment for children? To the include in the manuscript

**Response:
Samples from all suspected cases of BU including children who reported to the clinic were collected by healthcare professionals who are well-trained and knowledgeable about the clinical presentation of** BU.

10. Sample size is too small. The main limitation is the sample size.

**Response:
This sample size is adequate to provide robust estimates of specificity and clinically meaningful estimates of sensitivity under routine field conditions. Confidence intervals are reported to indicate the precision of all diagnostic accuracy estimates**.**The sample size for this diagnostic evaluation was determined to estimate the clinical sensitivity and specificity of the Mu-RPA assay conducted in a mobile suitcase laboratory, using IS2404 PCR as the reference standard. Sample size requirements were calculated with a single-proportion formula at a 95% confidence level. Based on previous evaluations of the Mu-RPA assay, we assumed an expected sensitivity of approximately 85% and aimed for a precision of ±10%. This resulted in a minimum requirement of 49 PCR-confirmed Buruli ulcer cases. For specificity, with an expected specificity of 95% and the same precision, we required at least 18 PCR-negative samples. Given the field-based nature of the study and the availability of clinically suspected cases at Buruli ulcer treatment centers, we were able to enrolled a total of 73 participants, consisting of 39 PCR-positive and 34 PCR-negative cases.**

11. If possible, include user feedback from healthcare workers on ease of use and acceptability.

**Response**

**We appreciate this suggestion. Formal user feedback from healthcare workers was not collected as part of the current study, as the assay was primarily performed by trained laboratory personnel rather than frontline healthcare workers. We have acknowledged this in the discussion and indicated that future studies will incorporate structured assessments of ease of use and acceptability among healthcare workers to further inform implementation and scale-up.**

12. Expand on reasons for lower sensitivity in FNA samples and propose solutions.

**Response
In the discussion, we addressed the observed lower sensitivity in FNA samples and examine potential contributing factors, including low bacterial load, sampling variability, and challenges related to specimen quality and DNA recovery. This discussion has been strengthened by proposing practical mitigation strategies, such as optimizing sampling techniques, diluting extracted DNA to reduce potential inhibitors associated with the crude extraction method, retaking samples when necessary and improving DNA extraction protocols by includin a field friendly a purification step.**

13. Discuss cost implications more thoroughly, feasibility of $10/test in endemic settings. Consider adding a cost-benefit analysis comparing this platform to PCR and other alternatives.

**Response:**

**We have enhanced the discussion to examine the cost implications of the RPA assay, particularly its feasibility at an estimated $10 per test in endemic, resource-limited settings. We also compare these factors to alternative field-friendly diagnostic options. Furthermore, we emphasize that, although the per-test cost is modest, successful broader implementation will necessitate careful consideration of supply chains, training, and integration into existing healthcare infrastructure key elements for ensuring sustainability and scalability in endemic regions.**

14. The sample size should be increased or adequately justified to strengthen the validity of the findings. Add limitations explicitly: small sample size, single-country evaluation, lack of multicenter validation. Include future directions: integration with WHO NTD roadmap, multiplexing potential, digital reporting tools.

**Response:**

**This have been addressed in the discussion.**

15. Correct typographical errors (e.g., “Sevety-three” → “Seventy-three”). Line 186

**Response:**

**This have been corrected.**

16. Use consistent terminology: “BU-RPA mobile laboratory platform” throughout.

**Response:**

**We have carefully revised the manuscript to ensure consistent use of the term “BU-RPA mobile laboratory platform”.**

17. The methodology section needs clearer description and justification of the approach used.

**Response:**

**We have revised the methodology section to offer a clearer and more detailed description of the study design, sample collection, DNA extraction, RPA assay workflow, and field implementation procedures. Furthermore, we have included explicit justification for our chosen approach, explaining why the RPA assay was selected, how it aligns with WHO priorities for field-deployable BU diagnostics, and the rationale for comparing it against the in-house IS2404 qPCR in the introduction.**

18. The discussion should be expanded to include limitations of the study and potential future applications of the findings.

**Response:**

**We have expanded the dicussion to include limitation and potential future applications of the findings**

19. References need a comprehensive review to ensure accuracy, consistency, and proper alignment with in-text citations.The references require a thorough review for accuracy and consistency. In the discussion section, the statement “Developing field-deployable diagnostic tools for neglected tropical diseases (NTDs) such as Buruli ulcer (BU) remains a key research priority for the World Health Organization (WHO) [1, 13]” implies that references 1 and 13 are WHO documents. However, in the reference list, these citations correspond to Yotsu RR et al. and Souza AA et al., respectively, and are not WHO sources. Please ensure that all in-text citations accurately reflect the referenced documents and revise either the text or the references to maintain consistency.

**Response:
We appreciate the reviewer for pointing out this inconsistency. We have carefully reviewed and revised all references to ensure accuracy and consistency between in-text citations and the reference list. Specifically, we have corrected the cited statement in the discussion to accurately reflect the nature of the referenced documents. Additionally, we have revised the text as needed to avoid implying WHO authorship for non-WHO sources. All citations now correctly correspond to their respective publications.**

Reviewer #3:

The manuscript describes the clinical validation of an isothermal assay (Mu-RPA) for the detection of M. ulcerans DNA using the specific IS2404 target. Specifically, they were using a field friendly assay setup that was split over two portable suitcases including both DNA extraction and amplification/read-out. Analysing different clinical samples from suspect BU patients, the authors found a sensitivity of this RPA assay of 83 % compared to the diagnostic gold standard of PCR detection and even up to 91 % when testing swabs. This kind of clinical validation in field settings is a crucial step towards the application of point-of-care testing platforms. The successful application of the relatively new RPA technology is an exciting finding. However, the scientific rigor could be increased, by including additional controls and sample size calculations. Further, statistical analyses should be included to support the findings and conclusions (major revision). Overall, advancing molecular diagnostic methods towards field applicability is not only of interest to researchers in the field but due to the potential to translate to other pathogens also of value for other researchers.

20. The hypothesis and specific objectives could be stated more clearly in the end of the introduction. What did the authors expect as outcome, which outcome measures were focussed on?

**Response:
The hypothesis, specific objectives, and measurable outcomes are clearly outlined in the introduction**.

21. A description of the study setting is missing (prevalence, incidence) and could be included in the introduction.

**Response:
We have included a brief description of the study setting in the methods section.**

22. Where the PCR-negative patients diagnosed with other diseases?

**Response:**

**The PCR-negative patients were diagnosed as tropical ulcers with unknown aetiology, and were referred for routine wound management as standard practice.**

23. Could the authors explain why no healthy controls were included?

**Response:
This study did not include healthy controls. In our previous research, we assessed the analytical specificity of the Mu-RPA assay and confirmed its 100% specificity for detecting Mycobacterium ulcerans [1]. Furthermore, we implemented quality control measures in this study, such as extraction controls and no-template controls, to monitor contamination and minimize false-positive results in field conditions. The current study was specifically designed to evaluate the mobile platform performance in individuals clinically suspected of having BU, focusing on real-world applicability rather than reassessing analytical specificity with healthy controls**.

24. No sample size calculation was included. As no prevalence data is included, it cannot be assessed whether the sample size is sufficient.

**Response:
The sample size for this diagnostic evaluation was determined to estimate the clinical sensitivity and specificity of the Mu-RPA assay conducted in a mobile suitcase laboratory, using IS2404 PCR as the reference standard. Sample size requirements were calculated with a single-proportion formula at a 95% confidence level. Based on previous evaluations of the Mu-RPA assay, we assumed an expected sensitivity of approximately 85% and aimed for a precision of ±10%. This resulted in a minimum requirement of 49 PCR-confirmed Buruli ulcer cases. For specificity, with an expected specificity of 95% and the same precision, we required at least 18 PCR-negative samples. Given the field-based nature of the study and the availability of clinically suspected cases at Buruli ulcer treatment centers, we enrolled a total of 73 participants, consisting of 39 PCR-positive and 34 PCR-negative cases. This sample size was adequate to provide robust estimates of specificity and clinically meaningful estimates of sensitivity under routine field conditions. Confidence intervals are reported to indicate the precision of all diagnostic accuracy estimates**.

25. The statistical analyses are not outlined in the methods section. The authors should add this to the methods section, as well as a sample size calculation to motivate the number of analysed samples.

**Response:**

**Statistical analyses has been included in the method section.**

26. Could the authors expand on how informed consent was obtained from children and illiterate persons, if applicable?

**Response:**

**Informed consent was obtained from all participants prior to enrolment. For illiterate participants, study information was read aloud in the local language mostly in Twi/Fante, and consent was documented by thumbprint in the presence of an impartial witness. For children, written consent was obtained from parents or legal guardians, and age-appropriate assent was obtained from the children when applicable. Participation was voluntary, with the right to withdraw at any time.**

27. Please include the positivity cutoff of the PCR and RPA and how it was determined for the RPA assay.

**Response:**

**Positivity threshold cut of PCR was 36 cycles perfomed on BioRad PCR machine. RPA amplfication was done with Axxin T8-ISO fluorometer (Axxin Pty Ltd., Victoria, Australia). We programmed the T8-fluorometer using the T8-ISO Desktop application (Axxin Pty Ltd, Victoria, Australia) based on our earlier study [1] to identify the lowest copies of IS2404 DNA sequence of *M.ulcerans* that effectively distinguished positive samples from negative controls, based on serial dilutions of a molecular standard. All RPA tests were conducted at 42 °C for 900 seconds (15 minutes), with mixing occurring after 4 minutes. A sample was considered positive if it exhibited either an amplitude or gradient of at least 900 mV over a 40-second sliding window during the amplification phase of the test (from 300 to 900 seconds) [1].**

28. What is the limit of detection of this assay?

**Response:**

**The limit of detection of MU-RPA assay is 45 copies of the IS2404 sequence of M. ulcerans, along with a high diagnostic sensitivity of 88% and a specificity of 100% from our previous study [1]**

29. Were the samples tested in triplicates? If not, please motivate why.

**Response:**

**All samples were tested once (in singlicate) due to resource and financial constraints.**

30. Was a non-template control included, how was controlled for contamination?

**Response:**

**Non-template and extraction controls were included in each assay to monitor potential contamination. Workstations were thoroughly cleaned before and after each procedure, gloves were changed frequently between work areas, and dedicated pipettes and consumables were used for each workstation, with no sharing of equipment between suitcases. In addition, the suitcases were positioned with the backs of the lids facing each other to further minimize the risk of cross-contamination.**

31. For the comparison of turnaround time between PCR and RPA there is no statistical test performed and no standard deviation in indicated.

**Response:**

**Turn arround time Between RPA and PCR was not compared as the differences were clear.**

32. Did the authors also check whether samples that were stored in the buffer shorter or longer than the average time are more or less positive for M. ulcerans DNA detection?

**Response:**

**The effect of sample storage duration in buffer on PCR or RPA positivity was not formally assessed. For RPA, samples were rapidly eluted into buffer and DNA extraction was performed immediately, minimizing potential effects of storage time.**

33. Was the significance of the influence of type of sample or BU lesion tested with a statistical test? Please add in the methods section which test was used and what the cutoff for hypothesis acceptance was.

**Response:**

**The statistical tests used for comparing turnaround time of RPA of the sample types and lesion types are stated in the methods section and the figure legend.**

34. I suggest adding a comparison of the positivity of PCR and RPA, to understand whether the observed RPA assay sensitivity is significantly different to the gold standard (paired testing).

**Response:**

**We appreciate the reviewer's suggestion. A direct comparison of PCR and RPA positivity is already included in the contingency table (Table 2), which presents paired results for both assays. This table facilitates the assessment of concordance and discordance between PCR (the reference standard) and the RPA assay, allowing for the interpretation of RPA sensitivity in relation to PCR. Therefore, we did not conduct an additional analysis of positivity, as the necessary information is already available and clearly presented.**

35. Figure 1 + 2 could be merged in a general figure and reduced to Figures 1F, 2A (with measurements?), 2C, and 2B.

**Response:**

**We appreciate the reviewer's suggestion and have modified the figures according.**

36. Figure 3 could be developed further, I recommend depicting the workflow in the different suitcases in more detail. As is, the figure does not have much additional value to the text.

**Response:**

**We appreciate the reviewer’s suggestion. To improve clarity while maintaining visual readability, we have expanded the figure legend (now Figure 2) to describe the workflow in greater detail. Adding more text directly to the figure would have made it visually cluttered; therefore, we opted to enhance the explanatory information in the legend instead..**

37. Figure 4: It could be helpful to include the sample number per condition in the figure

**Response:**

**The number of sample per conditions have been added to the figure 3 (previously figure 4) legend.**

38. Table 3: for FNA, should the sensitivity not be 32/39 = 82 %? The table could benefit from adding a line of total positives and negatives (for swab, FNA, and total), to better understand where the sensitivity calculations come from.

**Response:**

**We thank the reviewer for highlighting this issue. The sensitivity calculation for FNA samples has been corrected accordingly. In addition, Table 3 has been revised to include totals for positive and negative samples for swabs, FNAs, and the overall dataset, thereby improving transparency and clarity of the sensitivity calculations.**

39. Some of the patients diagnosed by the PCR assay were not detected by the BU-RPA. The authors mention that a higher limit of detection could be the reason. This should be put in the context of previously determined LODs with this assay and also other reasons should be considered, e.g. DNA extraction efficiency that could be influenced by the thick mycobacterial cell wall.

**Response**

**We have enhanced the discussion to examine the cost implications of the RPA assay, particularly its feasibility at an estimated $10 per test in endemic, resource-limited settings. We also compare these factors with alternative field-friendly diagnostic options. Furthermore, we emphasize that although the per-test cost is relatively low, successful broader implementation will necessitate careful consideration of supply chains, training, and integration into existing healthcare infrastructure critical components for ensuring sustainability and scalability in endemic regions.**

40. A further explanation why the FNA sample matrix may be detrimental to the detection efficiency would be helpful. Swabs were only taken from ulcerating lesions, could there be a relationship of the increased sensitivity with higher numbers of bacteria in this type of lesion? How could the sensitivity of FNA samples be increased?

**Response**

**The discussion addresses the potential negative impact of the FNA sample matrix on detection efficiency, considering factors such as low bacterial load, the presence of inhibitory substances, and difficulties in DNA recovery from aspirates. We emphasize that swab samples were taken exclusively from ulcerative lesions, which typically contain higher bacterial loads, likely resulting in the increased sensitivity observed in swab-based detection. Additionally, we explore strategies to enhance the sensitivity of FNA samples, including optimized sampling techniques, repeat sampling, dilution of extracted DNA to mitigate inhibitors, and improved DNA extraction methods. These factors have been highlighted as crucial areas for future research.**

41. Please elaborate if the other assays BU-LAMP and fTLC were also done on swabs and FNAs.

**Response**

**The previously reported BU-LAMP and fTLC assays were conducted on swab from ulcerative lesions, and FNA from non ulcerative lesions. This has been stated in the discussion.**

42. Could the authors include whether the reagents used in the DNA extraction need special storage conditions or are they stable at ambient temperature?

**Response**

**The reagents used for DNA extraction in the BU-RPA mobile laboratory platform remain stable under ambient field conditions throughout typical deployment periods and do not require cold-chain storage [2]. This stability enhances the platform's feasibility for use in resource-limited and remote settings.**

43. The authors mention that no internal positive control and quantification was included. Could they expand on why this was left out and how it can be confidentially determined that the negative results in the BU-RPA were not due to extraction or assay failure?

**Response**

**Although the current field implementation did not include an internal positive control or quantification module, assay reliability was maintained through the use of non-template and extraction controls. These controls facilitate confident interpretation of negative results by monitoring for contamination or assay failure. The decision to omit an internal control was aimed to simplify the field workflow, but future implementations could benefit from the inclusion of such controls to further enhance assay confidence.**

44. If samples are not collected in the hospital but more remotely, could the time between sample collection and start of extraction influence the result? Would it be possible to perform the test on inactivated samples as well (e.g. with ethanol, due to biosafety considerations)?

**Response
We recognize that delays in processing samples collected from remote locations may affect detection due to DNA degradation. However, in this study, all samples were collected and processed as promptly as possible after collection in the hospitals for BU-RPA platform. Only samples for PCR was collected and transported to the reference laboratory for testing. Furthermore, using inactivated samples (e.g., preserved in ethanol) for BU-RPA testing may improve biosafety for field staff; this approach warrants further validation in future studies.**

References

1.	Frimpong M, Ahor HS, Wahed AAE, Agbavor B, Sarpong FN, Laing K, et al. Rapid detection of Mycobacterium ulcerans with isothermal recombinase polymerase amplification assay. PLoS Negl Trop Dis. 2019;13(2):e0007155. Epub 20190201. doi: 10.1371/journal.pntd.0007155. PubMed PMID: 30707706; PubMed Central PMCID: PMCPMC6373974.

2.	Frimpong M, Ahor HS, Sakyi SA, Agbavor B, Akowuah E, Phillips RO. Rapid Extraction Method of Mycobacterium ulcerans DNA from Clinical Samples of Suspected Buruli Ulcer Patients. Diagnostics (Basel). 2019;9(4). Epub 20191126. doi: 10.3390/diagnostics9040204. PubMed PMID: 31779247; PubMed Central PMCID: PMCPMC6963521.
